# Supplementary material for: Three-dimensional molecular architecture of mouse organogenesis
Source: Nat Commun. 2023 Jul 31;14:4599. doi: 10.1038/s41467-023-40155-7 (PMC10390492; doi:10.1038/s41467-023-40155-7)
Supplement: Supplementary file 1 — Supplementary Information [file 41467_2023_40155_MOESM1_ESM.pdf]

Supplementary Information

Three-dimensional molecular architecture of mouse organogenesis

Fangfang Qu[1,2,3,9], Wenjia Li[1,3,4,9], Jian Xu[1,9], Ruifang Zhang[1], Jincan Ke[5], Xiaodie Ren[1], Xiaogao Meng[5,6], Lexin Qin[5], Jingna Zhang[1], Fangru Lu[1], Xin Zhou[1], Xi Luo[5], Zhen Zhang[5], Minhan Wang[5], Guangming Wu[1,3,7], Duanqing Pei[8], Jiekai Chen[1,5], Guizhong Cui[1,3,7,*], Shengbao Suo[3,4,*], Guangdun Peng[1,5,*]

[1]Center for Cell Lineage and Atlas, Bioland Laboratory, Guangzhou

[2]GMU-GIBH Joint School of Life Sciences, The Guangdong-Hong Kong-Macau Joint Laboratory for Cell Fate Regulation and Diseases, Guangzhou Medical University, Guangzhou, Guangdong 510005, China

[3]Guangzhou Laboratory, Guangzhou, Guangdong 510005, China

[4]The First Affiliated Hospital of Guangzhou Medical University, State Key Laboratory of Respiratory Disease, Guangzhou, Guangdong 510005, China

[5]Center for Cell Lineage and Development, CAS Key Laboratory of Regenerative Biology, Guangdong Provincial Key Laboratory of Stem Cell and Regenerative Medicine, GIBH-HKU Guangdong-Hong Kong Stem Cell and Regenerative Medicine Research Centre, Guangzhou Institutes of Biomedicine and Health, University of the Chinese Academy of Sciences, Chinese Academy of Sciences, Guangzhou 510530, China

[6]Life Science and Medicine, University of Science and Technology of China, Hefei 230026, Anhui, China

[7]School of Basic Medical Sciences, Guangzhou Medical University, Guangzhou, Guangdong 510005, China

[8]Laboratory of Cell Fate Control, School of Life Sciences, Westlake University, Hangzhou, China

[9]These authors contributed equally to this work

*co-corresponding author:

cui_guizhong@gzlab.ac.cn

suo_shengbao@gzlab.ac.cn

peng_guangdun@gibh.ac.cn

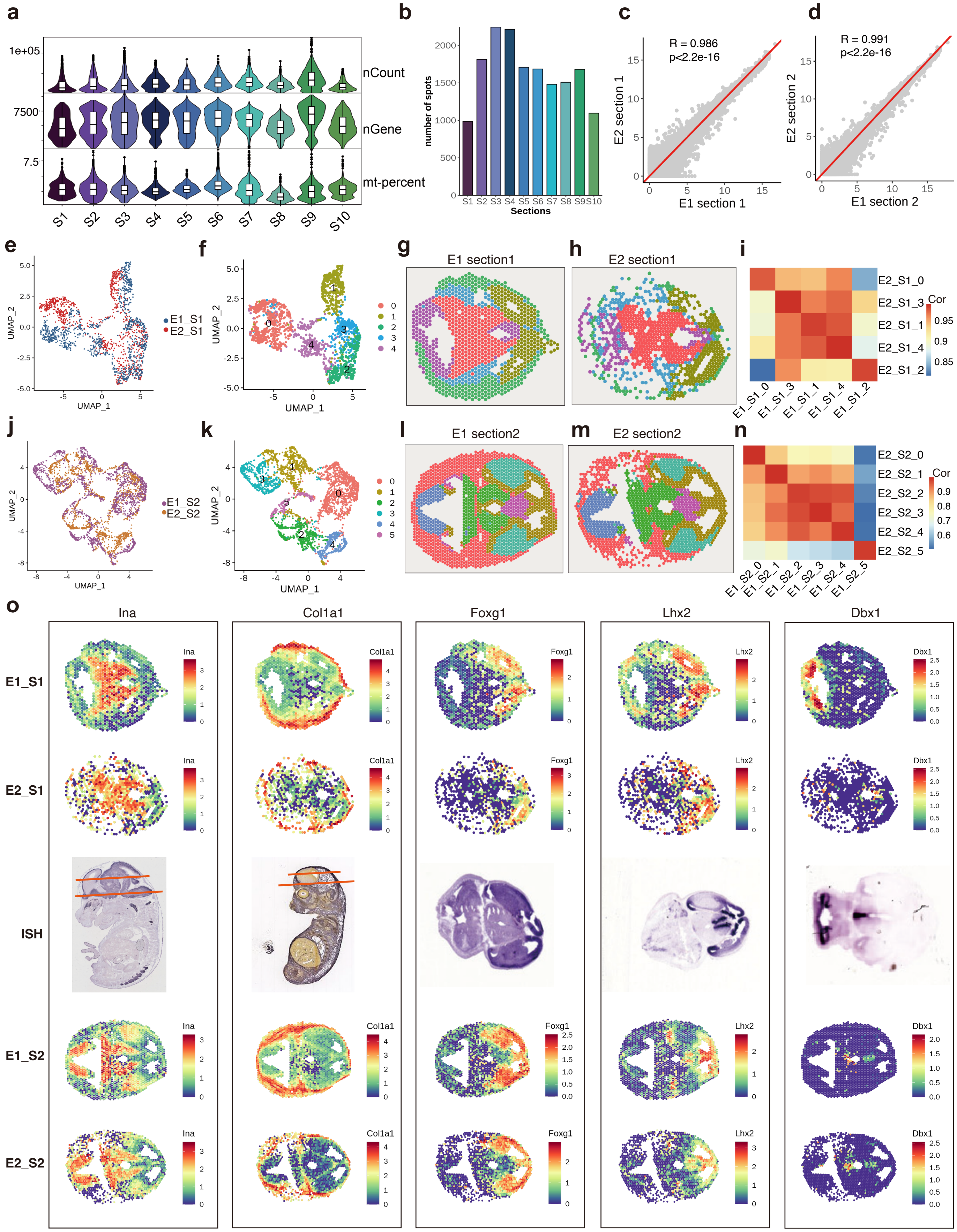
a
b
c
d
nCount
nGene
mt-percent
number of spots
Sections
R = 0.986
p<2.2e-16
E2 section 1
E1 section 1
R = 0.991
p<2.2e-16
E2 section 2
E1 section 2
e
f
g
h
i
E1 section1
E2 section1
E1_S1
E2_S1
UMAP_1
UMAP_2
Cor
j
k
l
m
n
E1 section2
E2 section2
E1_S2
E2_S2
o
Ina
Col1a1
Foxg1
Lhx2
Dbx1
ISH

Supplementary Fig.1| Data Quality and replicates of spatial transcriptional atlas for mouse embryo development in organogenesis at E13.5.

(**a**) Distribution of UMI count (upper), genes (middle) and mitochondrial percentage (bottom) for the collected 10 sections. n (from S1 to S10) = 986, 1813, 2244, 2214, 1708, 1686, 1482, 1508, 1681, 1096. The centerline of Violin plot indicates median; box limits are upper and lower quartiles; and the whiskers are highest and lowest values within 1.5 times interquartile range from the box. (**b**) The total number of spots in each tissue section included for downstream analyses. (**c**) Pearson correlation coefficient (R=0.986, p< 2.2e-16) of pseudo-bulk profiles for section 1 (S1) from mouse embryo 1 (E1) and similar section of embryo 2 (E2). Statistical significance was calculated by two-sided T-test. (**d**) Pearson correlation coefficient (R= 0.991, p< 2.2e-16) of pseudo-bulk profiles for section 2 (S2) from E1 and similar section from E2. Statistical significance was calculated by two-sided T-test. (**e,f**) UMAP embedding of spots from S1 of E1 and E2, colored by sample identities (**e**) and clustering of spatial regions (**f**). Colors represent cluster assignment based on Louvain clustering of all spots from the two sections. (**g,h**) 5 clustered spatial regions showed on tissue section1 of E1 (**g**) and E2 (**h**). The same color scheme as (**f**) for spot colors. (**i**)Heatmap showing pairwise spatial cluster correlations of section1 of E1 and E2. (**j,k**) UMAP embedding of spots from S2 of E1 and E2, colored by sample identities (**j**) and clustering of spatial regions (**k**). (**l,m**) 6 clustered spatial regions showed on tissue section2 of E1 (**l**) and E2 (**m**). (**n**)Heatmap showing pairwise spatial cluster correlations of section2 of E1 and E2.(**O**) Spatial visualization of expression for selected marker genes (*Ina*, *Col1a1*, *Foxg1*, *Lhx2* and *Dbx1*) of the S1 and S2 from two embryos (E1 and E2) and ISH images of the related genes from MGI database and Allen Brain Atlas. Source data are provided as a Source Data file.

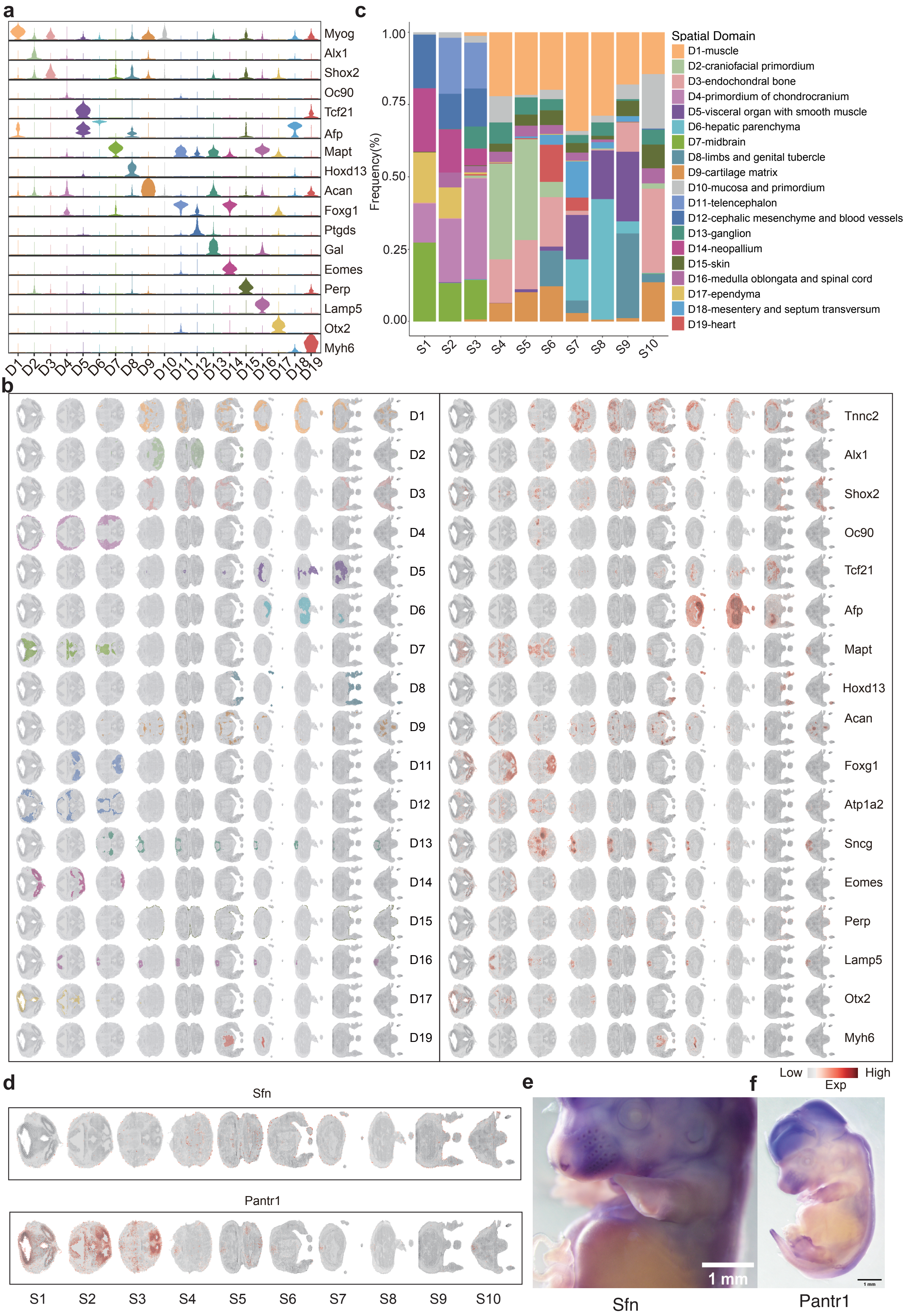

a
Myog
Alx1
Shox2
Oc90
Tcf21
Afp
Mapt
Hoxd13
Acan
Foxg1
Ptgds
Gal
Eomes
Perp
Lamp5
Otx2
Myh6
D1 D2 D3 D4 D5 D6 D7 D8 D9 D10 D11 D12 D13 D14 D15 D16 D17 D18 D19
c
Frequency(%)
1.00
0.75
0.50
0.25
0.00
S1 S2 S3 S4 S5 S6 S7 S8 S9 S10
Spatial Domain
D1-muscle
D2-craniofacial primordium
D3-endochondral bone
D4-primordium of chondrocranium
D5-visceral organ with smooth muscle
D6-hepatic parenchyma
D7-midbrain
D8-limbs and genital tubercle
D9-cartilage matrix
D10-mucosa and primordium
D11-telencephalon
D12-cephalic mesenchyme and blood vessels
D13-ganglion
D14-neopallium
D15-skin
D16-medulla oblongata and spinal cord
D17-ependyma
D18-mesentery and septum transversum
D19-heart
b
D1
D2
D3
D4
D5
D6
D7
D8
D9
D11
D12
D13
D14
D15
D16
D17
D19
Tnnc2
Alx1
Shox2
Oc90
Tcf21
Afp
Mapt
Hoxd13
Acan
Foxg1
Atp1a2
Sncg
Eomes
Perp
Lamp5
Otx2
Myh6
d
Sfn
Pantr1
S1 S2 S3 S4 S5 S6 S7 S8 S9 S10
e
f
Low
High
Exp
1 mm
Sfn
Pantr1

Supplementary Fig.2| Spatial domains and the signature genes at E13.5.

(**a**) Stacked violin plots showing the expression levels of the specific markers in each of the spatial domain. (**b**) Highlighted spatial domains mapping across all the ten sections (left) and the spatial expression of specific marker genes for each spatial domain across all the ten sections (right). (**c**) Percentage of spatial domains assigned in each section. (**d,e,f**) spatial expression of *Sfn* and *Pantr1* across 10 sections of ST data (**d**) and whole mount in situ hybridization (WISH) showing expression pattern of *Sfn* (**e**, n = 6) and *Pantr1* (**f**, n = 3) in E13.5 embryo. Scale bars,1 mm.

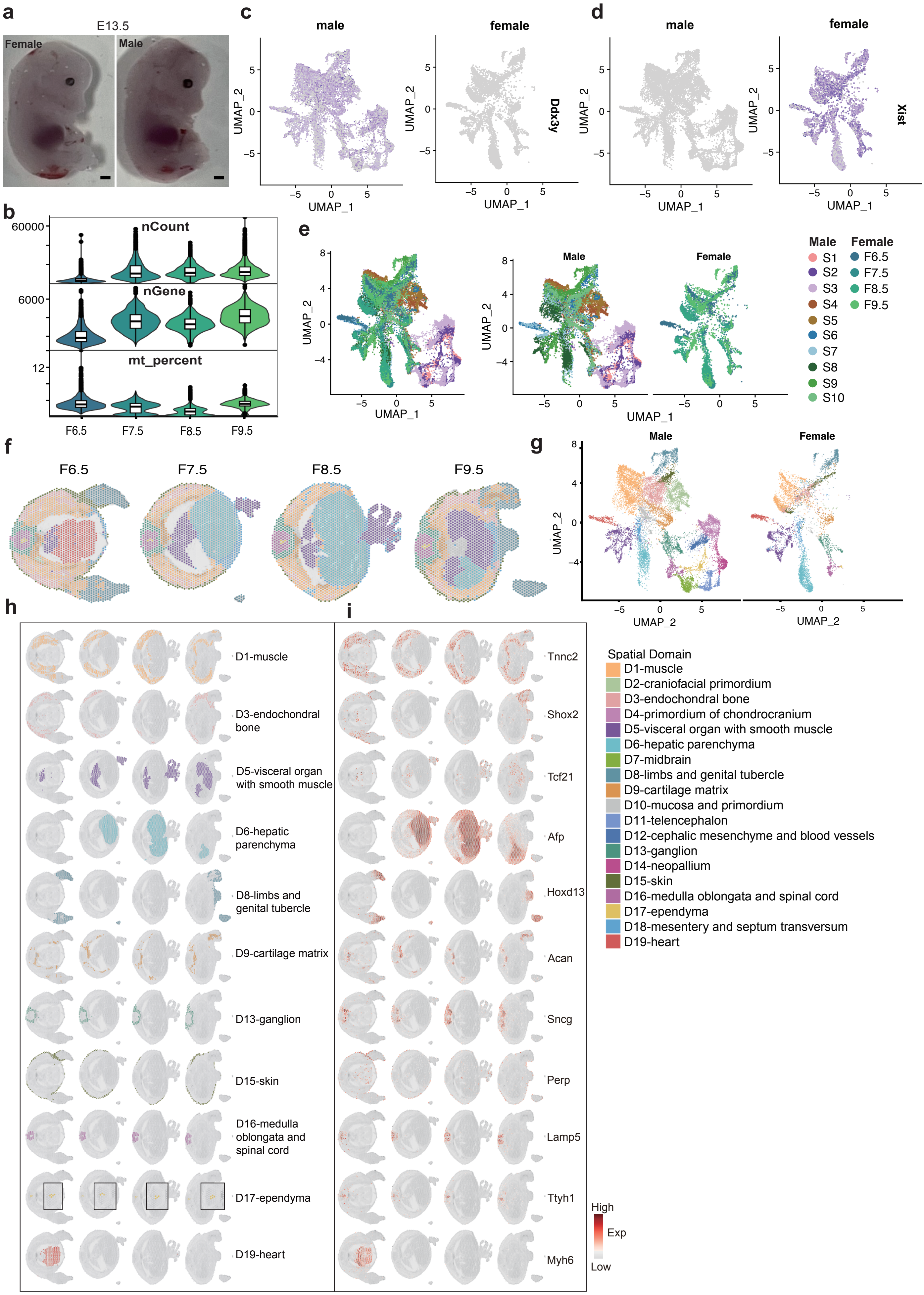

a
E13.5
Female
Male
b
nCount
nGene
mt_percent
F6.5
F7.5
F8.5
F9.5
c
male
female
Ddx3y
d
male
female
Xist
UMAP_1
UMAP_2
e
Male
Female
S1
S2
S3
S4
S5
S6
S7
S8
S9
S10
F6.5
F7.5
F8.5
F9.5
f
F6.5
F7.5
F8.5
F9.5
g
Male
Female
h
D1-muscle
D3-endochondral bone
D5-visceral organ with smooth muscle
D6-hepatic parenchyma
D8-limbs and genital tubercle
D9-cartilage matrix
D13-ganglion
D15-skin
D16-medulla oblongata and spinal cord
D17-ependyma
D19-heart
i
Tnnc2
Shox2
Tcf21
Afp
Hoxd13
Acan
Sncg
Perp
Lamp5
Ttyh1
Myh6
High
Exp
Low
Spatial Domain
D1-muscle
D2-craniofacial primordium
D3-endochondral bone
D4-primordium of chondrocranium
D5-visceral organ with smooth muscle
D6-hepatic parenchyma
D7-midbrain
D8-limbs and genital tubercle
D9-cartilage matrix
D10-mucosa and primordium
D11-telencephalon
D12-cephalic mesenchyme and blood vessels
D13-ganglion
D14-neopallium
D15-skin
D16-medulla oblongata and spinal cord
D17-ependyma
D18-mesentery and septum transversum
D19-heart

Supplementary Fig.3| Spatial transcriptome of female replicate embryo at E13.5.

(**a**) Representative images of E13.5 female (n = 4) and male (n = 6) embryos. Scale bar=1mm. (**b**) Distribution of UMI count (upper), genes (middle) and mitochondrial percentage (bottom) for the collected 4sections. n (from F6.5 to F9.5) =1577, 1407, 1768, 1651. The centerline of Violin plot indicates median; box limits are upper and lower quartiles; and the whiskers are highest and lowest values within 1.5 times interquartile range from the box. (**c,d**) UMAP plot showing the sex specific gene expression *Ddx3y* (**c**) and *Xist* (**d**) in male or female embryo. (**e,f,g**) UMAP embedding of spots from male embryo E1 and female embryo E3 colored by sections, either grouped or split by sex (**e**) and colored by spatial domains (**g**). The spatial distribution of predicted spatial domain in embryo E3 across 4 tissue sections according to label transfer from E1 (**f**). (**h,i**) Highlighted spatial domains mapping across four sections (**h**) and the spatial expression of specific marker genes for each spatial domain (**i**) in E3. Source data are provided as a Source Data file.

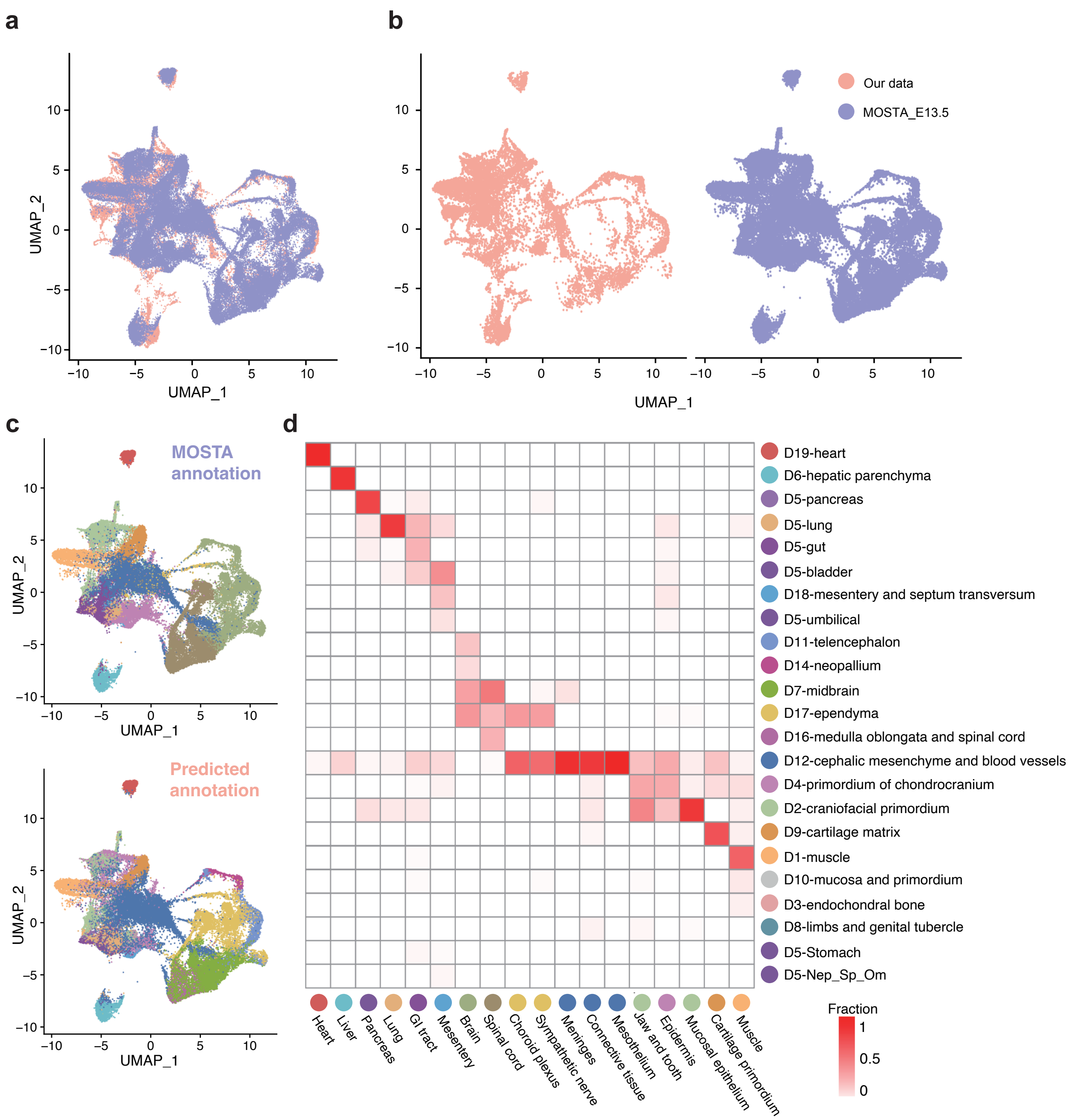

a
b
c
d
UMAP_1
UMAP_2
Our data
MOSTA_E13.5
MOSTA annotation
Predicted annotation
D19-heart
D6-hepatic parenchyma
D5-pancreas
D5-lung
D5-gut
D5-bladder
D18-mesentery and septum transversum
D5-umbilical
D11-telencephalon
D14-neopallium
D7-midbrain
D17-ependyma
D16-medulla oblongata and spinal cord
D12-cephalic mesenchyme and blood vessels
D4-primordium of chondrocranium
D2-craniofacial primordium
D9-cartilage matrix
D1-muscle
D10-mucosa and primordium
D3-endochondral bone
D8-limbs and genital tubercle
D5-Stomach
D5-Nep_Sp_Om
Heart
Liver
Pancreas
Lung
GI tract
Mesentery
Brain
Spinal cord
Choroid plexus
Sympathetic nerve
Meninges
Connective tissue
Mesothelium
Jaw and tooth
Epidermis
Mucosal epithelium
Cartilage primordium
Muscle
Fraction
1
0.5
0

Supplementary Fig.4| Integrative analysis of spatial transcriptomes from different experiments

(**a,b,c**) UMAP plot of the integrated spatial data of E13.5 section 1 of MOSTA dataset and 10 sections from E1 grouped (**a**) or split (**b**) by the data source, and colored by MOSTA annotation (**c**) or by the predicted annotation through label transferring from E1. (**d**) Comparison of predicted spatial domain and MOSTA annotation. Heatmap showing the percentage of predicted spatial domain in each of MOSTA annotated cluster.

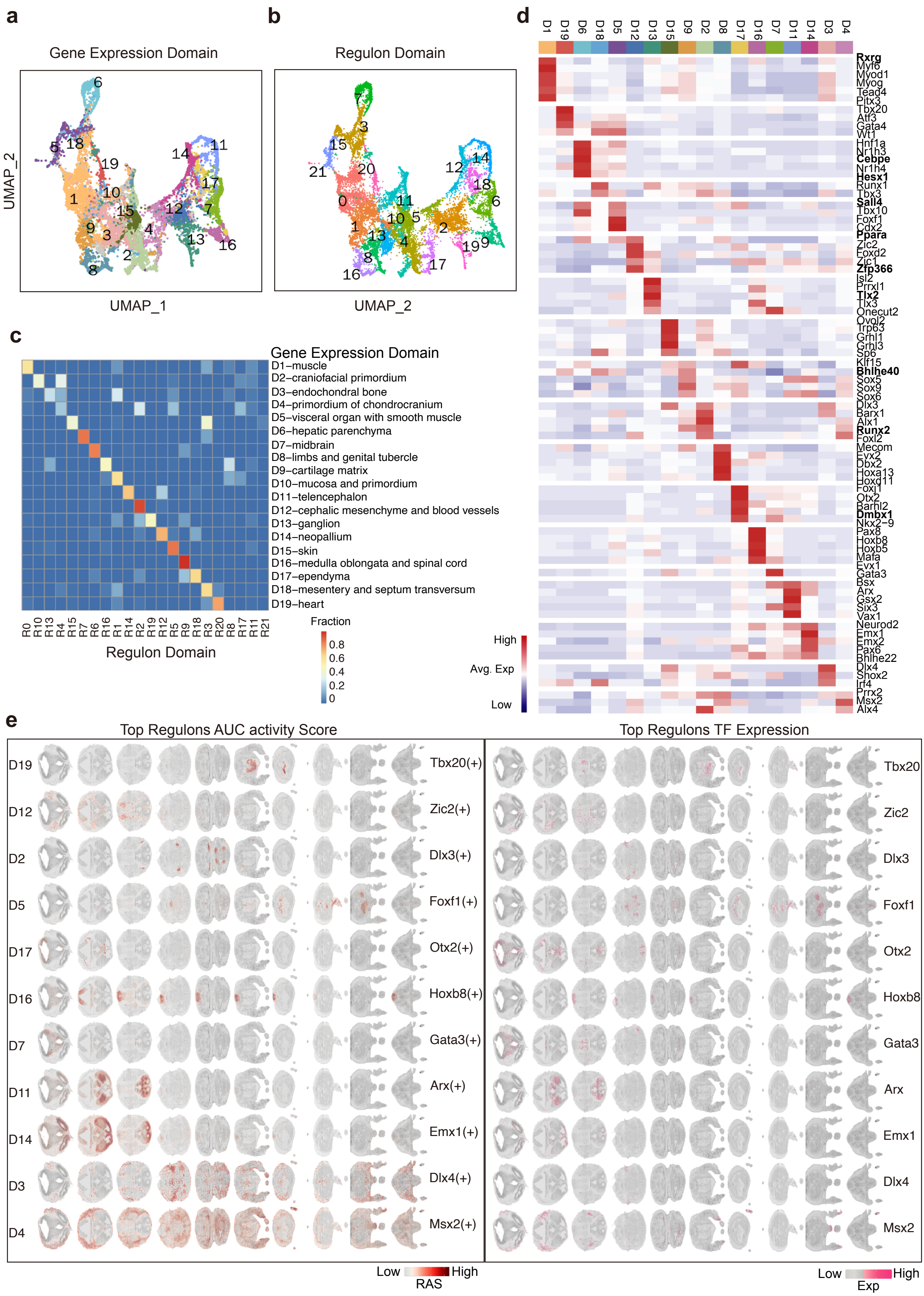

a
Gene Expression Domain
UMAP_2
UMAP_1
b
Regulon Domain
UMAP_2
c
Gene Expression Domain
D1–muscle
D2–craniofacial primordium
D3–endochondral bone
D4–primordium of chondrocranium
D5–visceral organ with smooth muscle
D6–hepatic parenchyma
D7–midbrain
D8–limbs and genital tubercle
D9–cartilage matrix
D10–mucosa and primordium
D11–telencephalon
D12–cephalic mesenchyme and blood vessels
D13–ganglion
D14–neopallium
D15–skin
D16–medulla oblongata and spinal cord
D17–ependyma
D18–mesentery and septum transversum
D19–heart
R0 R10 R13 R4 R15 R7 R6 R16 R1 R14 R2 R19 R12 R5 R9 R18 R3 R20 R8 R17 R11 R21
Regulon Domain
Fraction
0.8
0.6
0.4
0.2
0
d
D1 D19 D6 D18 D5 D12 D13 D15 D9 D2 D8 D17 D16 D7 D11 D14 D3 D4
Rxrg
Myf6
Myod1
Myog
Tead4
Pitx3
Tbx20
Atf3
Gata4
Wt1
Hnf1a
Nr1h3
Cebpe
Nr1h4
Hesx1
Runx1
Tbx3
Sall4
Tbx10
Foxf1
Cdx2
Ppara
Zic2
Foxd2
Zic1
Zfp366
Isl2
Prrxl1
Tlx2
Tlx3
Onecut2
Ovol2
Trp63
Grhl1
Grhl3
Sp6
Klf15
Bhlhe40
Sox5
Sox9
Sox6
Dlx3
Barx1
Alx1
Runx2
Foxl2
Mecom
Evx2
Dbx2
Hoxa13
Hoxd11
Foxj1
Otx2
Barhl2
Dmbx1
Nkx2-9
Pax8
Hoxb8
Hoxb5
Mafa
Evx1
Gata3
Bsx
Arx
Gsx2
Six3
Vax1
Neurod2
Emx1
Emx2
Pax6
Bhlhe22
Dlx4
Shox2
Irf4
Prrx2
Msx2
Alx4
High
Avg. Exp
Low
e
Top Regulons AUC activity Score
Top Regulons TF Expression
D19 D12 D2 D5 D17 D16 D7 D11 D14 D3 D4
Tbx20(+)
Zic2(+)
Dlx3(+)
Foxf1(+)
Otx2(+)
Hoxb8(+)
Gata3(+)
Arx(+)
Emx1(+)
Dlx4(+)
Msx2(+)
Tbx20
Zic2
Dlx3
Foxf1
Otx2
Hoxb8
Gata3
Arx
Emx1
Dlx4
Msx2
Low
High
RAS
Low
High
Exp

Supplementary Fig.5| Gene regulation network in the spatial domains.

(**a,b**) UMAP embedding of all spots based on regulon activity scores (RAS). Colors represent spatial domain assignment based on gene expressions (**a**) or RAS (**b**). (**c**) Concordance between spatial domains based on gene expression and RAS. The color bar of heatmap represents the percentage of spots in spatial domains (rows) that was labeled as the indicated regulon clusters (each row of proportions was summed to 1). (**d**) Heatmap of mean TF expression in each spatial domain corresponding to the selected top regional specific regulons in Fig. 2a and relatively new TFs in corresponding domains were marked in bold. (**e**) Spatial distribution of RAS on tissues for selected specific regulons and related TFs in the spatial domains.

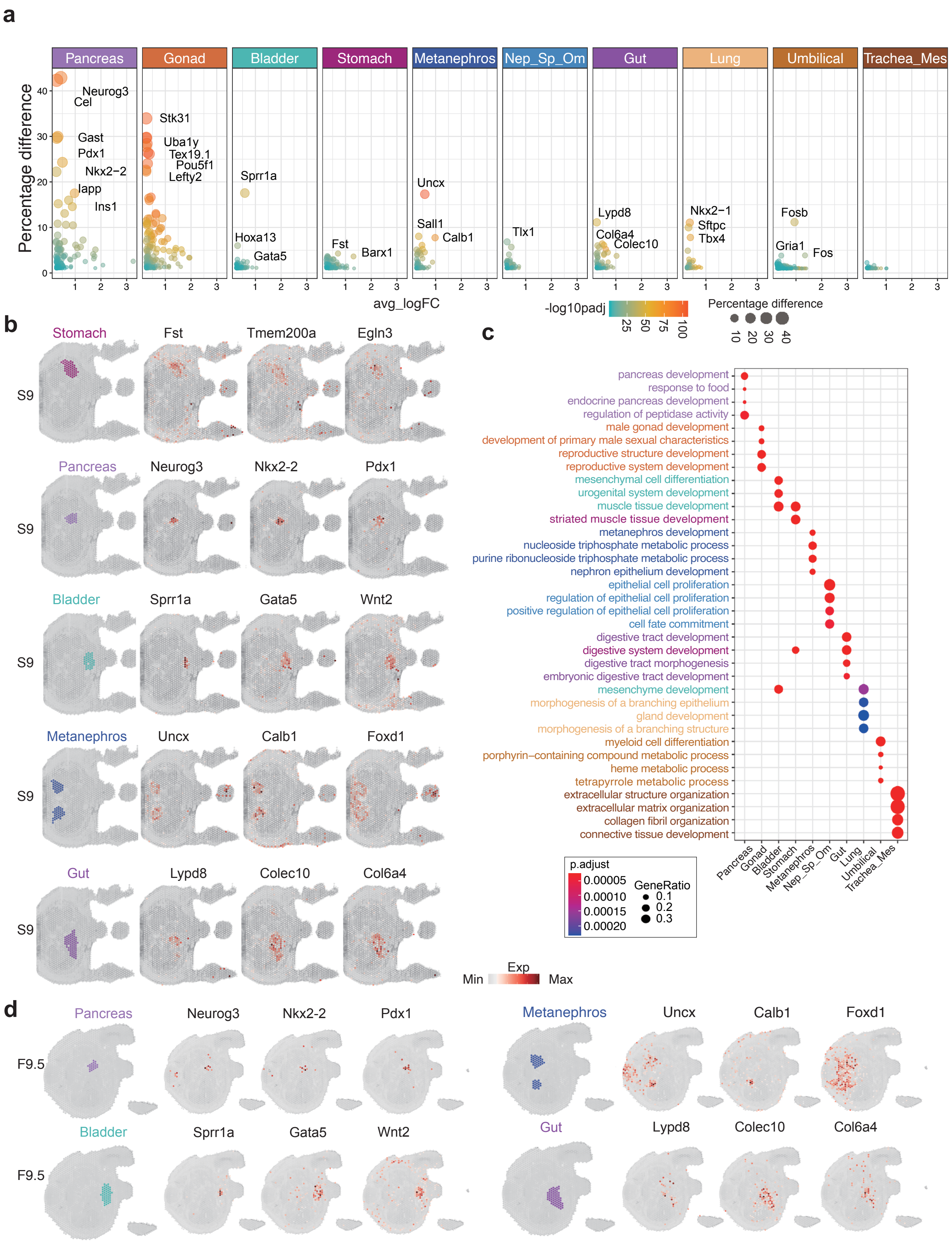

a
Pancreas
Gonad
Bladder
Stomach
Metanephros
Nep_Sp_Om
Gut
Lung
Umbilical
Trachea_Mes
Percentage difference
avg_logFC
Neurog3
Cel
Gast
Pdx1
Nkx2−2
Iapp
Ins1
Stk31
Uba1y
Tex19.1
Pou5f1
Lefty2
Sprr1a
Hoxa13
Gata5
Fst
Barx1
Uncx
Sall1
Calb1
Tlx1
Lypd8
Col6a4
Colec10
Nkx2−1
Sftpc
Tbx4
Fosb
Gria1
Fos
-log10padj
25 50 75 100
Percentage difference
10 20 30 40
b
Stomach
Fst
Tmem200a
Egln3
S9
Pancreas
Neurog3
Nkx2-2
Pdx1
Bladder
Sprr1a
Gata5
Wnt2
Metanephros
Uncx
Calb1
Foxd1
Gut
Lypd8
Colec10
Col6a4
Exp
Min
Max
c
pancreas development
response to food
endocrine pancreas development
regulation of peptidase activity
male gonad development
development of primary male sexual characteristics
reproductive structure development
reproductive system development
mesenchymal cell differentiation
urogenital system development
muscle tissue development
striated muscle tissue development
metanephros development
nucleoside triphosphate metabolic process
purine ribonucleoside triphosphate metabolic process
nephron epithelium development
epithelial cell proliferation
regulation of epithelial cell proliferation
positive regulation of epithelial cell proliferation
cell fate commitment
digestive tract development
digestive system development
digestive tract morphogenesis
embryonic digestive tract development
mesenchyme development
morphogenesis of a branching epithelium
gland development
morphogenesis of a branching structure
myeloid cell differentiation
porphyrin−containing compound metabolic process
heme metabolic process
tetrapyrrole metabolic process
extracellular structure organization
extracellular matrix organization
collagen fibril organization
connective tissue development
Pancreas
Gonad
Bladder
Stomach
Metanephros
Nep_Sp_Om
Gut
Lung
Umbilical
Trachea_Mes
p.adjust
0.00005
0.00010
0.00015
0.00020
GeneRatio
0.1
0.2
0.3
d
Pancreas
Neurog3
Nkx2-2
Pdx1
F9.5
Metanephros
Uncx
Calb1
Foxd1
Bladder
Sprr1a
Gata5
Wnt2
Gut
Lypd8
Colec10
Col6a4

Supplementary Fig.6| Domain specific gene expression and regulation for visceral organs

(**a**) Scatter plots showing the statistical summary of differential expressed genes in each subclusters. X axis indicates the log2 foldchange of average expression between given spatial subcluster and all others in D5 domain, Y axis and the size of dots indicates the difference of percentage of gene expression detected between given subclusters and all others, and colored by -log10(p-adjust). Top different genes were indicated. Statistical significance was calculated by two-sided Wilcoxon rank sum test with Bonferroni correction. (**b**) Spatial distribution of subclusters and spatial expression of selected marker genes on different tissues in embryo E1. (**c**) Enriched GO terms of differential marker gene in each subcluster. The statistical analysis was performed by over-representation test and P value was adjusted by Benjamini-Hochberg method. Color bar indicated the adjusted p-value and dots are scaled by the gene ratio. (**d**) Spatial distribution of subclusters and spatial expression of selected marker genes on different tissues in embryo E3.

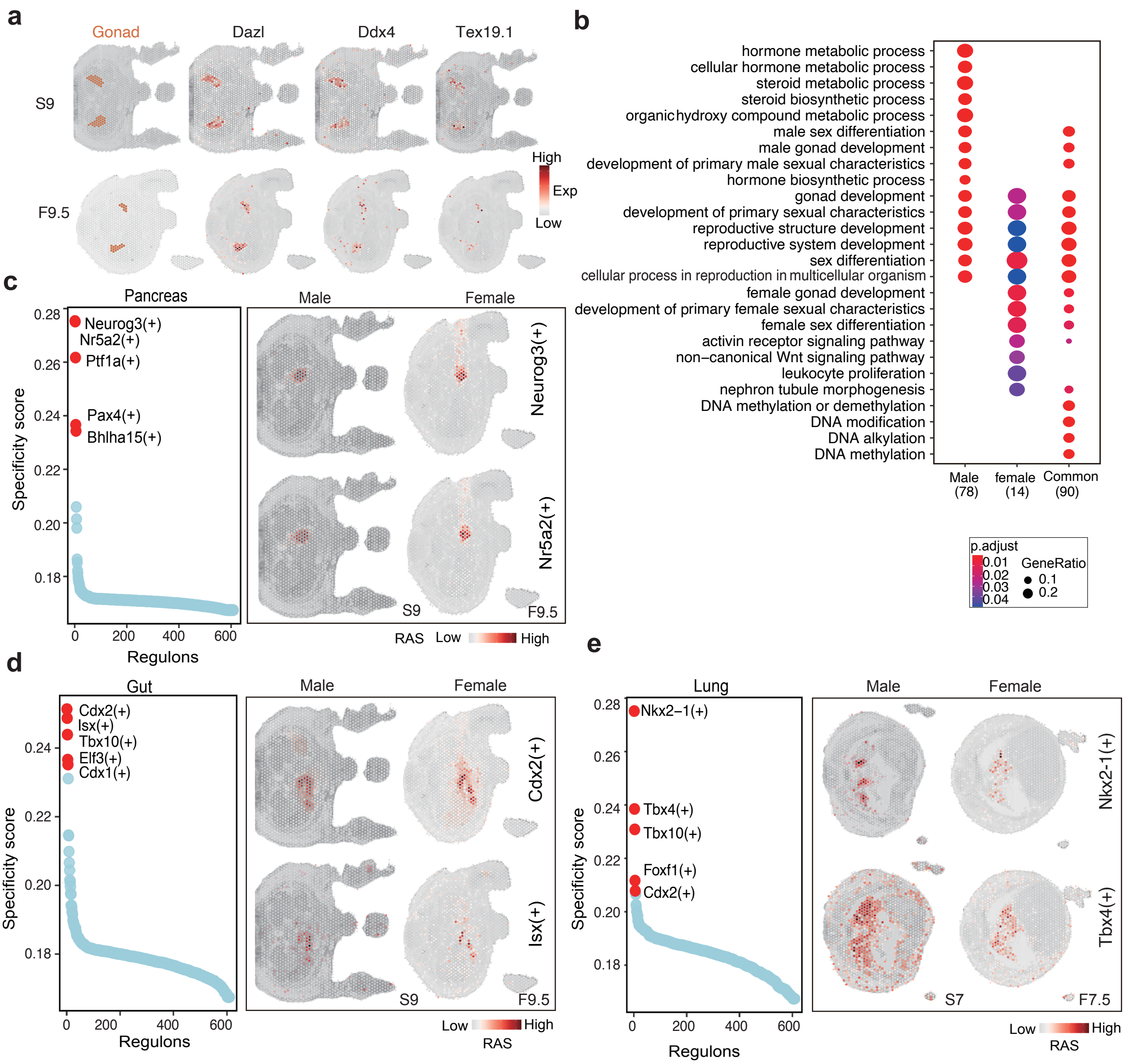
a
Gonad
Dazl
Ddx4
Tex19.1
S9
F9.5
High
Exp
Low
b
hormone metabolic process
cellular hormone metabolic process
steroid metabolic process
steroid biosynthetic process
organic hydroxy compound metabolic process
male sex differentiation
male gonad development
development of primary male sexual characteristics
hormone biosynthetic process
gonad development
development of primary sexual characteristics
reproductive structure development
reproductive system development
sex differentiation
cellular process in reproduction in multicellular organism
female gonad development
development of primary female sexual characteristics
female sex differentiation
activin receptor signaling pathway
non−canonical Wnt signaling pathway
leukocyte proliferation
nephron tubule morphogenesis
DNA methylation or demethylation
DNA modification
DNA alkylation
DNA methylation
Male (78)
female (14)
Common (90)
p.adjust
0.01
0.02
0.03
0.04
GeneRatio
0.1
0.2
c
Pancreas
Neurog3(+)
Nr5a2(+)
Ptf1a(+)
Pax4(+)
Bhlha15(+)
Specificity score
Regulons
Male
Female
Neurog3(+)
Nr5a2(+)
S9
F9.5
RAS
Low
High
d
Gut
Cdx2(+)
Isx(+)
Tbx10(+)
Elf3(+)
Cdx1(+)
Specificity score
Regulons
Male
Female
Cdx2(+)
Isx(+)
S9
F9.5
Low
High
RAS
e
Lung
Nkx2−1(+)
Tbx4(+)
Tbx10(+)
Foxf1(+)
Cdx2(+)
Specificity score
Regulons
Male
Female
Nkx2-1(+)
Tbx4(+)
S7
F7.5
Low
High
RAS

Supplementary Fig.7| Spatial characterization of sex specific gene expression in Gonad and gene regulation in major organs

(**a**) Spatial distribution of Gonad and common gonad marker genes on sections of both sexes. (**b**) Enriched GO terms of sex specific or common gonad genes. The statistical analysis was performed by over-representation test and P value was adjusted by Benjamini-Hochberg method. Color bar indicated the adjusted p-value and dots are scaled by the gene ratio. (**c,d,e**) Rank of regulons for Pancreas (**c**), Gut (**d**) and Lung (**e**) based on regulon specificity score (RSS) in E1 dataset (left) and spatial visualization of the activity score of top regulons in both E1 (right top) and replicate E3 (right bottom).

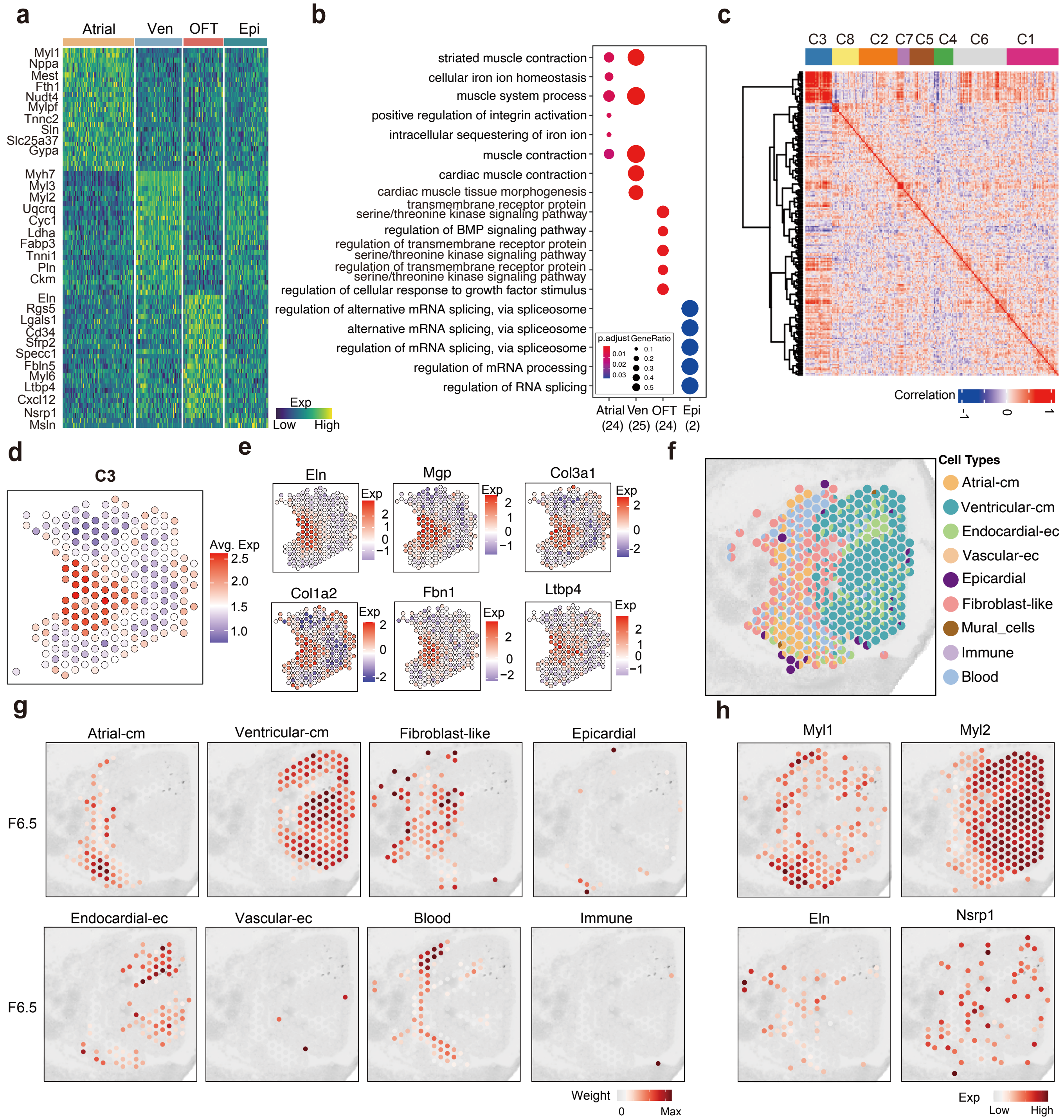

a
Atrial
Ven
OFT
Epi
Myl1
Nppa
Mest
Fth1
Nudt4
Mylpf
Tnnc2
Sln
Slc25a37
Gypa
Myh7
Myl3
Myl2
Uqcrq
Cyc1
Ldha
Fabp3
Tnni1
Pln
Ckm
Eln
Rgs5
Lgals1
Cd34
Sfrp2
Specc1
Fbln5
Myl6
Ltbp4
Cxcl12
Nsrp1
Msln
Exp
Low
High
b
striated muscle contraction
cellular iron ion homeostasis
muscle system process
positive regulation of integrin activation
intracellular sequestering of iron ion
muscle contraction
cardiac muscle contraction
cardiac muscle tissue morphogenesis
transmembrane receptor protein serine/threonine kinase signaling pathway
regulation of BMP signaling pathway
regulation of transmembrane receptor protein serine/threonine kinase signaling pathway
regulation of transmembrane receptor protein serine/threonine kinase signaling pathway
regulation of cellular response to growth factor stimulus
regulation of alternative mRNA splicing, via spliceosome
alternative mRNA splicing, via spliceosome
regulation of mRNA splicing, via spliceosome
regulation of mRNA processing
regulation of RNA splicing
p.adjust
GeneRatio
Atrial (24)
Ven (25)
OFT (24)
Epi (2)
c
C3
C8
C2
C7
C5
C4
C6
C1
Correlation
-1
0
1
d
C3
Avg. Exp
2.5
2.0
1.5
1.0
e
Eln
Mgp
Col3a1
Col1a2
Fbn1
Ltbp4
Exp
f
Cell Types
Atrial-cm
Ventricular-cm
Endocardial-ec
Vascular-ec
Epicardial
Fibroblast-like
Mural_cells
Immune
Blood
g
Atrial-cm
Ventricular-cm
Fibroblast-like
Epicardial
F6.5
Endocardial-ec
Vascular-ec
Blood
Immune
F6.5
Weight
0
Max
h
Myl1
Myl2
Eln
Nsrp1
Exp
Low
High

Supplementary Fig.8| Spatial gene analysis of heart subregions.

(**a**) Expression heatmap of top representative marker genes in heart subregions. (**b**) Enriched GO terms of differential marker gene in heart subregions. The statistical analysis was performed by over-representation test and P value was adjusted by Benjamini-Hochberg method. Color bar indicated the adjusted p-value and dots are scaled by the gene ratio. (**c,d,e**) Unsupervised spatial expression clustering of congenital heart diseases (CHD) genes revealed that genes in Cluster 3 were highly correlated (**c**). The representative spatial pattern of averaged expression of genes in Cluster 3 (**d**). Spatial distribution of selected genes in cluster 3 of CHD genes (**e**). (**f,g**) The spatial map of predicted cell types proportion in the heart region of section F6.5 in repeated embryo E3 and spatial visualization of deconvoluted weights of 8 heart specific cell types, except mural cells with a low proportion and a few of mapped spots. (**h**) Spatial expression of selected sub-domain specific marker genes *Myl1* for Atrial, *Myl2* for Ven, *Eln* for OFT, and *Nsrp1* for Epi in E3.

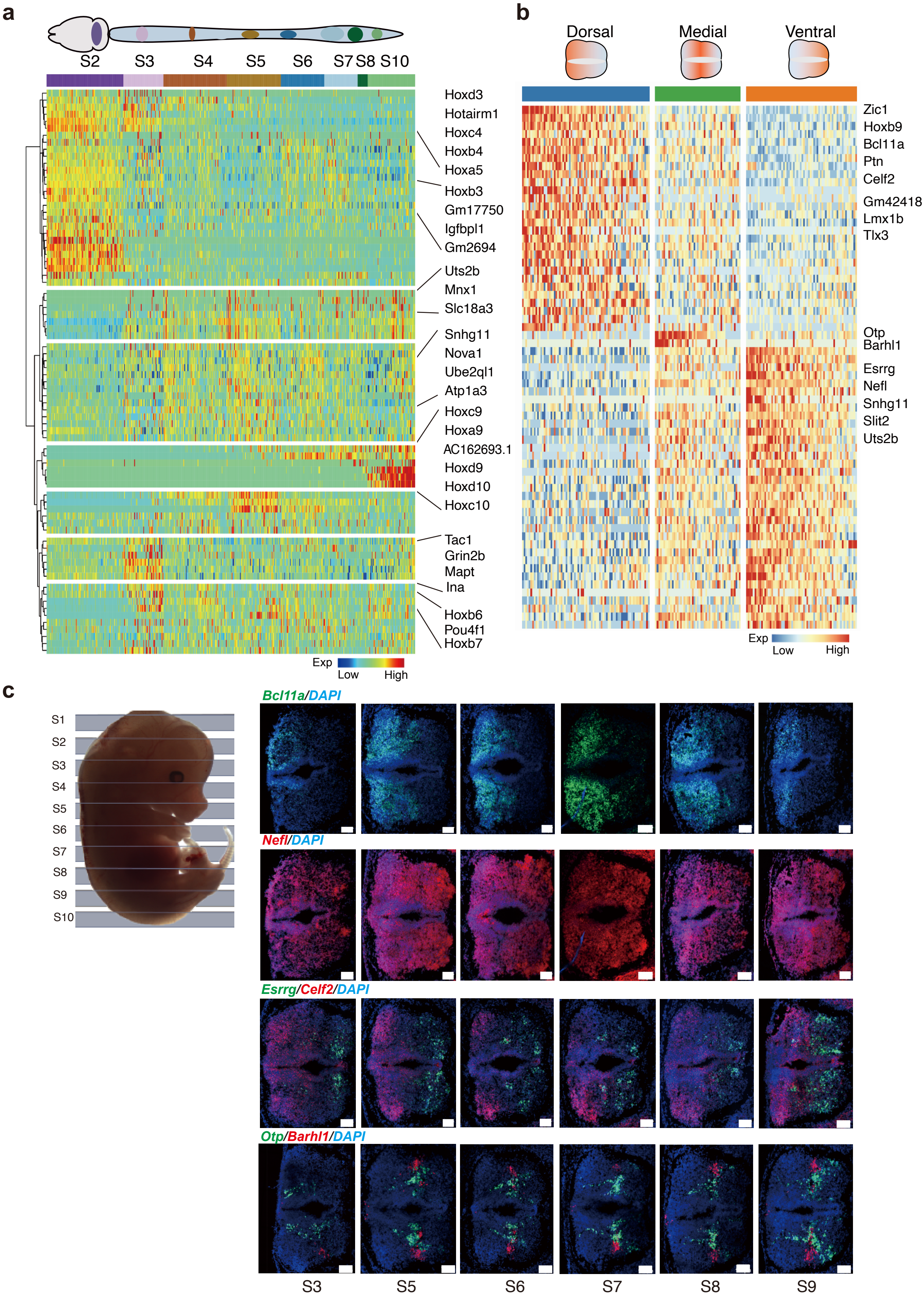
a
S2
S3
S4
S5
S6
S7
S8
S10
Hoxd3
Hotairm1
Hoxc4
Hoxb4
Hoxa5
Hoxb3
Gm17750
Igfbpl1
Gm2694
Uts2b
Mnx1
Slc18a3
Snhg11
Nova1
Ube2ql1
Atp1a3
Hoxc9
Hoxa9
AC162693.1
Hoxd9
Hoxd10
Hoxc10
Tac1
Grin2b
Mapt
Ina
Hoxb6
Pou4f1
Hoxb7
Exp
Low
High
b
Dorsal
Medial
Ventral
Zic1
Hoxb9
Bcl11a
Ptn
Celf2
Gm42418
Lmx1b
Tlx3
Otp
Barhl1
Esrrg
Nefl
Snhg11
Slit2
Uts2b
Exp
Low
High
c
S1
S2
S3
S4
S5
S6
S7
S8
S9
S10
Bcl11a/DAPI
Nefl/DAPI
Esrrg/Celf2/DAPI
Otp/Barhl1/DAPI
S3
S5
S6
S7
S8
S9

Supplementary Fig.9| Spatial patterning for spinal cord in mouse embryo organogenesis at E13.5.

(**a**) Heatmap showing the expression pattern of the identified A-P axis related genes in hindbrain and spinal cord from section 2 (S2) to section 10 (S10) along anterior to posterior. (**b**) Heatmap of differentially expressed genes in Dorsal (D), Medial (M), Ventral (V) regions of spinal cord along D-V axis. (**c**) RNAScope multiplex in situ hybridizations of D-V patterning genes in spinal cord, and representative images from hybridizations on serial sections from anterior to posterior ($n_{S5,6,7} = 3$, $n_{S3,8,9} = 2$). Scale bars, 100 μm.

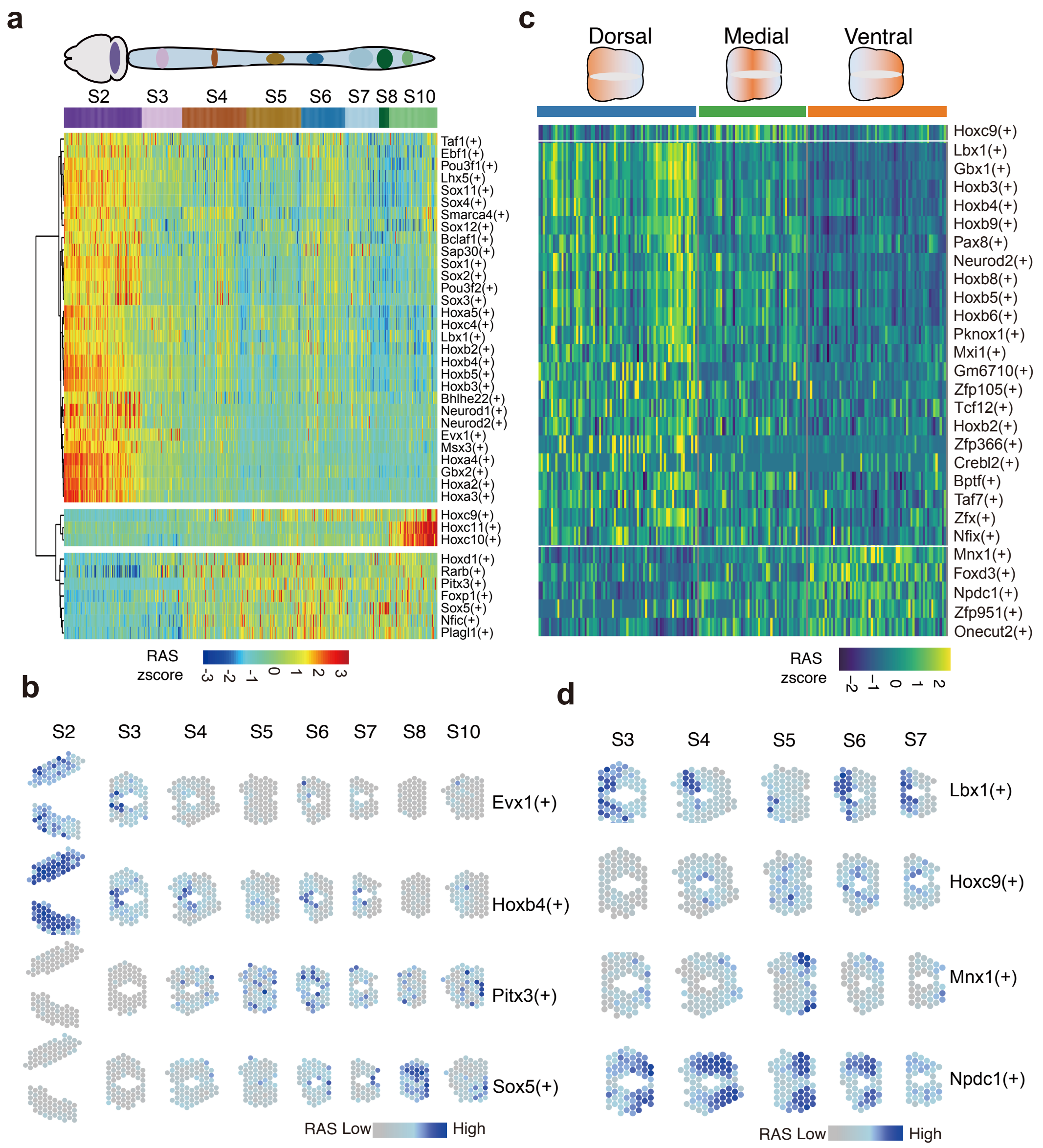
a
S2 S3 S4 S5 S6 S7 S8 S10
Taf1(+)
Ebf1(+)
Pou3f1(+)
Lhx5(+)
Sox11(+)
Sox4(+)
Smarca4(+)
Sox12(+)
Bclaf1(+)
Sap30(+)
Sox1(+)
Sox2(+)
Pou3f2(+)
Sox3(+)
Hoxa5(+)
Hoxc4(+)
Lbx1(+)
Hoxb2(+)
Hoxb4(+)
Hoxb5(+)
Hoxb3(+)
Bhlhe22(+)
Neurod1(+)
Neurod2(+)
Evx1(+)
Msx3(+)
Hoxa4(+)
Gbx2(+)
Hoxa2(+)
Hoxa3(+)
Hoxc9(+)
Hoxc11(+)
Hoxc10(+)
Hoxd1(+)
Rarb(+)
Pitx3(+)
Foxp1(+)
Sox5(+)
Nfic(+)
Plagl1(+)
RAS zscore
-3 -2 -1 0 1 2 3
c
Dorsal
Medial
Ventral
Hoxc9(+)
Lbx1(+)
Gbx1(+)
Hoxb3(+)
Hoxb4(+)
Hoxb9(+)
Pax8(+)
Neurod2(+)
Hoxb8(+)
Hoxb5(+)
Hoxb6(+)
Pknox1(+)
Mxi1(+)
Gm6710(+)
Zfp105(+)
Tcf12(+)
Hoxb2(+)
Zfp366(+)
Crebl2(+)
Bptf(+)
Taf7(+)
Zfx(+)
Nfix(+)
Mnx1(+)
Foxd3(+)
Npdc1(+)
Zfp951(+)
Onecut2(+)
RAS zscore
-2 -1 0 1 2
b
S2 S3 S4 S5 S6 S7 S8 S10
Evx1(+)
Hoxb4(+)
Pitx3(+)
Sox5(+)
RAS Low High
d
S3 S4 S5 S6 S7
Lbx1(+)
Hoxc9(+)
Mnx1(+)
Npdc1(+)
RAS Low High

Supplementary Fig.10| Spatial gene regulation in spinal cord patterning.

(**a**) Heatmap showing RAS of the identified A-P axis related regulons in hindbrain and spinal cord from section 2 to section 10 along anterior to posterior. (**b**) Spatial distribution of RAS of selected A-P axis related regulons in the hindbrain and spinal cord tissue spots across section 2 to 10. (**c**) Heatmap of detected D-V axis related regulons in Dorsal (D), Medial (M), Ventral (V) regions of spinal cord along D-V axis. (**d**) Spatial distribution of RAS of selected D-V axis related regulons in spinal cord tissue spots of section 3 to 7.

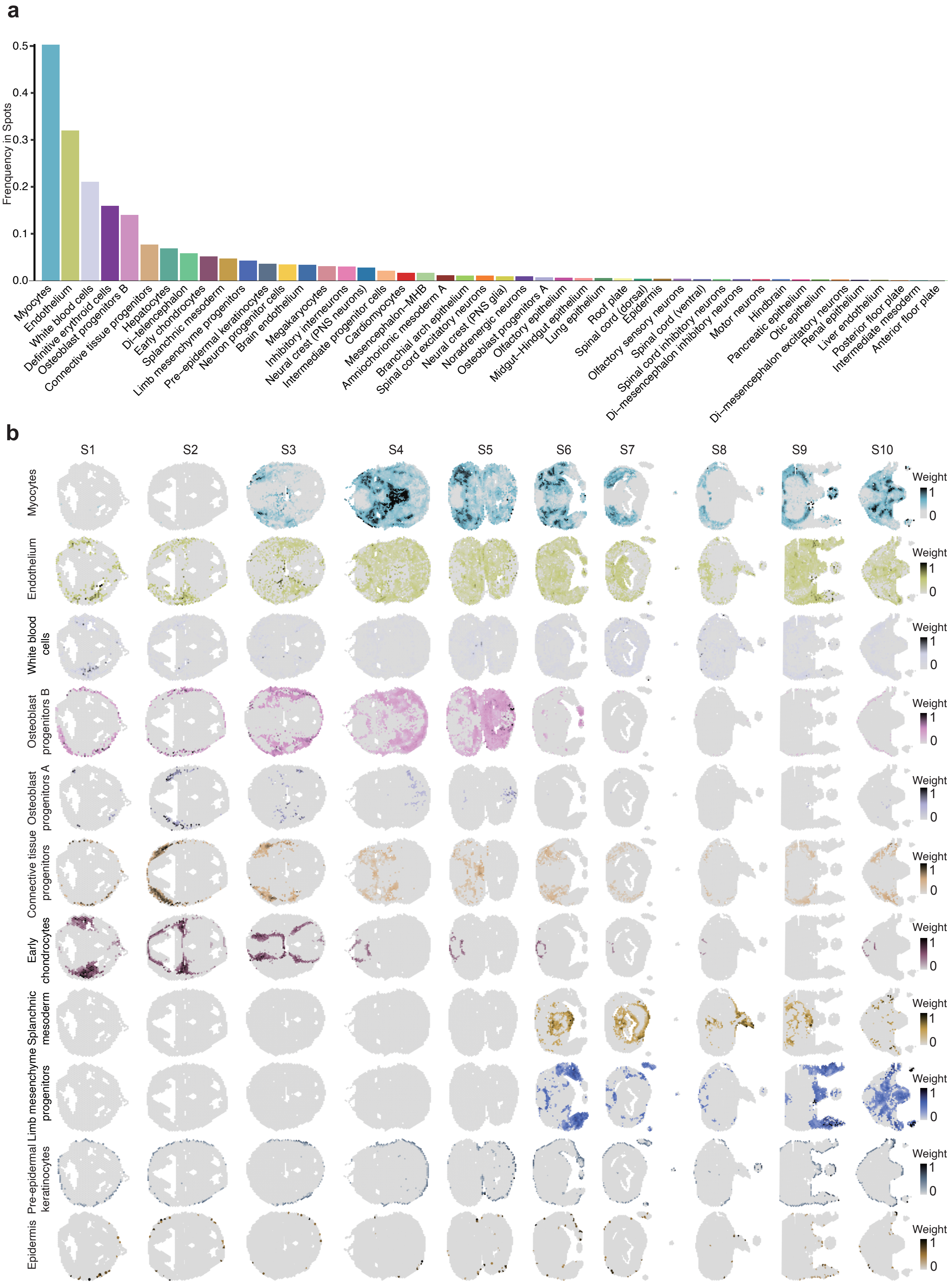
a
Frenquency in Spots
0.5
0.4
0.3
0.2
0.1
0.0
Myocytes
Endothelium
White blood cells
Definitive erythroid cells
Osteoblast progenitors B
Connective tissue progenitors
Hepatocytes
Di–telencephalon
Early chondrocytes
Splanchnic mesoderm
Limb mesenchyme progenitors
Pre–epidermal keratinocytes
Neuron progenitor cells
Brain endothelium
Megakaryocytes
Inhibitory interneurons
Neural crest (PNS neurons)
Intermediate progenitor cells
Cardiomyocytes
Mesencephalon–MHB
Amniochorionic mesoderm A
Branchial arch epithelium
Spinal cord excitatory neurons
Neural crest (PNS glia)
Noradrenergic neurons
Osteoblast progenitors A
Olfactory epithelium
Midgut–Hindgut epithelium
Lung epithelium
Roof plate
Spinal cord (dorsal)
Epidermis
Olfactory sensory neurons
Spinal cord (ventral)
Spinal cord inhibitory neurons
Di–mesencephalon inhibitory neurons
Motor neurons
Hindbrain
Pancreatic epithelium
Otic epithelium
Di–mesencephalon excitatory neurons
Renal epithelium
Liver endothelium
Posterior floor plate
Intermediate mesoderm
Anterior floor plate
b
S1
S2
S3
S4
S5
S6
S7
S8
S9
S10
Myocytes
Endothelium
White blood cells
Osteoblast progenitors B
Osteoblast progenitors A
Connective tissue progenitors
Early chondrocytes
Splanchnic mesoderm
Limb mesenchyme progenitors
Pre-epidermal keratinocytes
Epidermis
Weight
1
0

Supplementary Fig.11| Spatial mapping of cell types from mouse organogenesis at E13.5.

(**a**) Bar plot showing the proportion of cell types occupied in spots across all embryo tissue sections after mapping back to the spatial regions. (**b**) Spatial visualization of deconvoluted weights of 16 cell types. Source data are provided as a Source Data file.

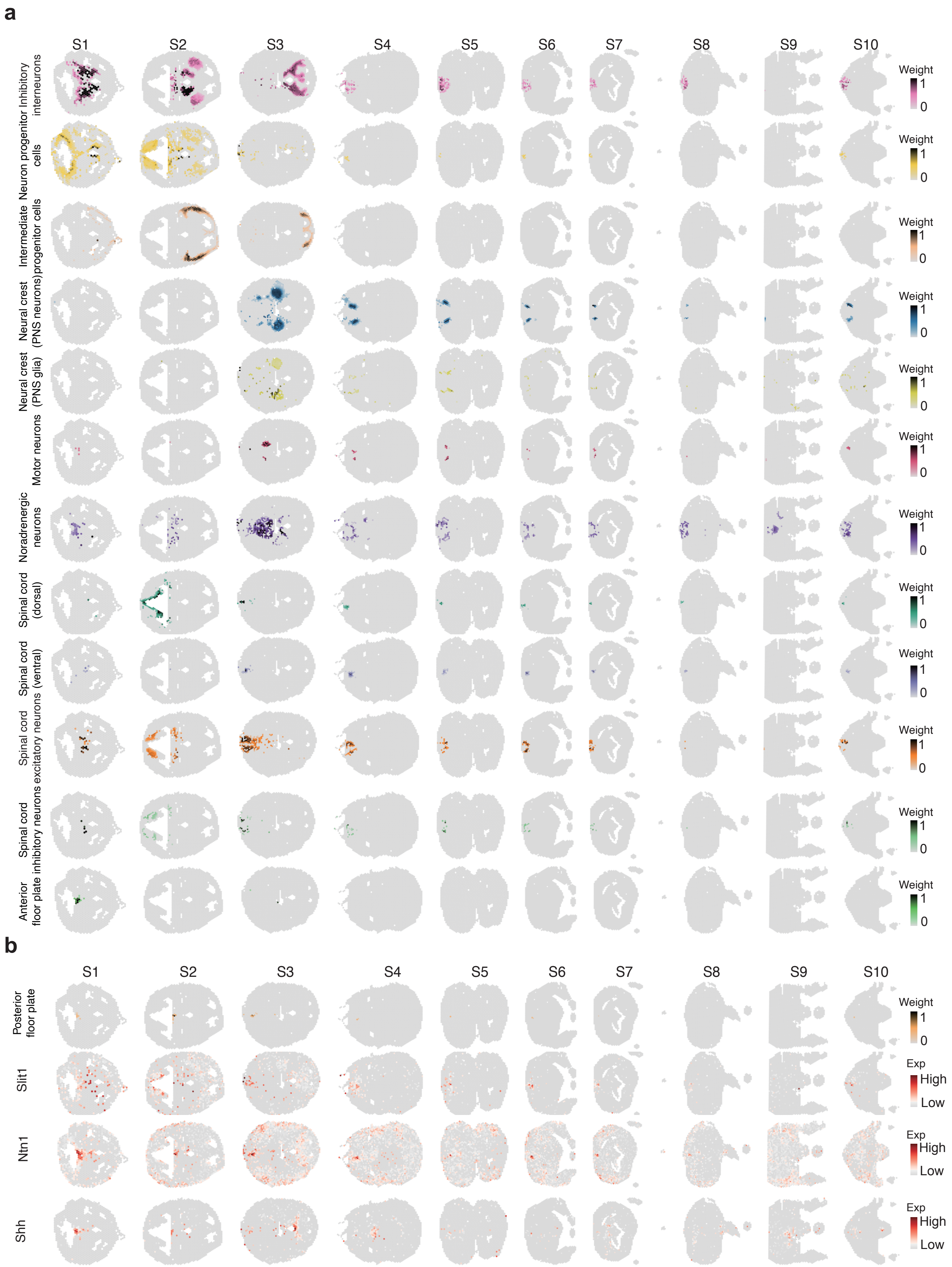
a
S1
S2
S3
S4
S5
S6
S7
S8
S9
S10
Inhibitory interneurons
Neuron progenitor cells
Intermediate progenitor cells
Neural crest (PNS neurons)
Neural crest (PNS glia)
Motor neurons
Noradrenergic neurons
Spinal cord (dorsal)
Spinal cord (ventral)
Spinal cord excitatory neurons
Spinal cord inhibitory neurons
Anterior floor plate
Weight
1
0
b
Posterior floor plate
Slit1
Ntn1
Shh
Exp
High
Low

Supplementary Fig.12| Cell type compositions in spatial domains across the embryo. (**a**) Spatial visualization of deconvoluted weights of neuron related cell types after spatial mapping. (**b**) Spatial visualization of deconvoluted weights of posterior floor plate cells and the expression of marker genes (*Slit1*, *Ntn1* and *Shh*) of posterior floor plate cells from TOME dataset across all embryo tissue sections.

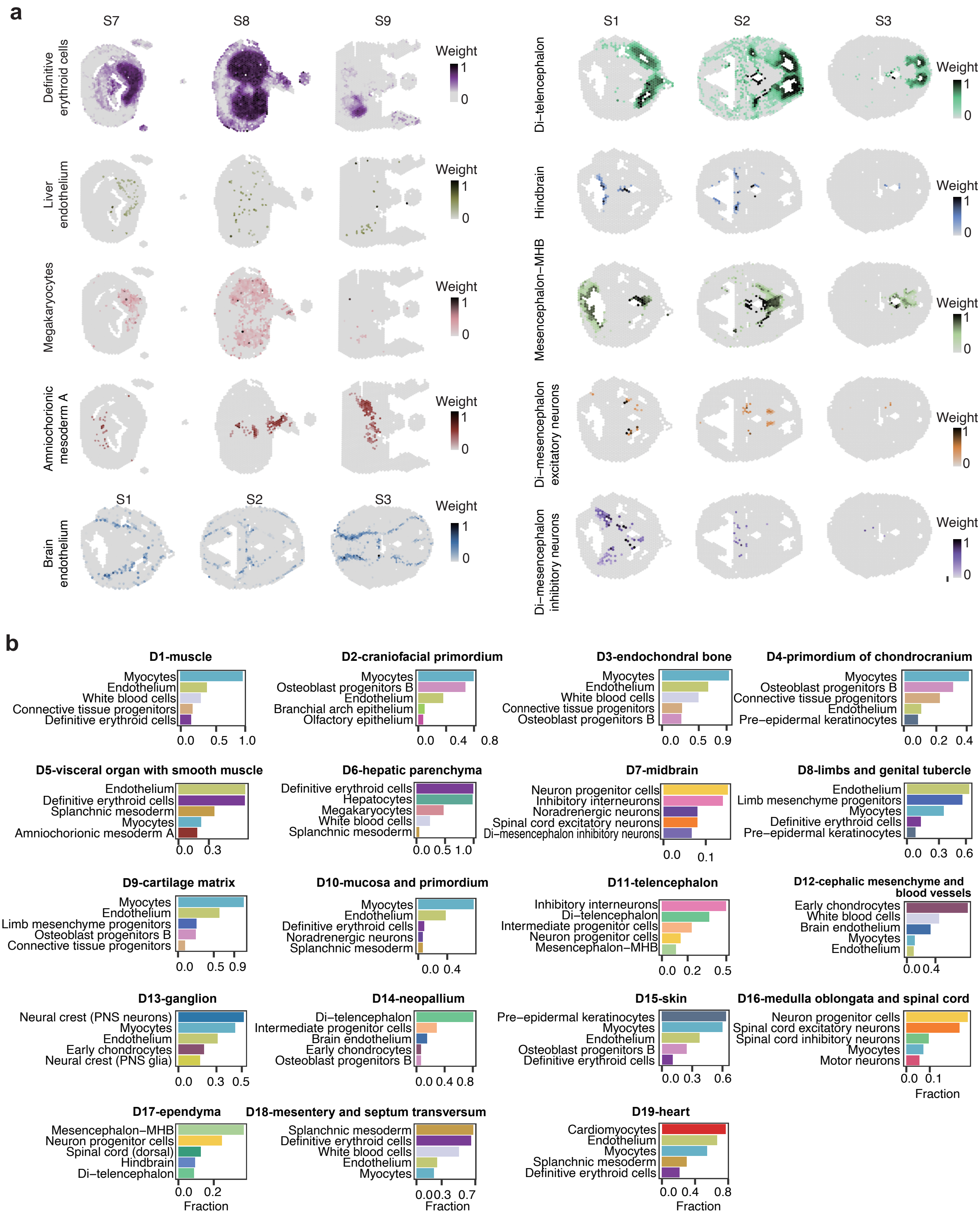
a
S7
S8
S9
S1
S2
S3
Definitive erythroid cells
Liver endothelium
Megakaryocytes
Amniochorionic mesoderm A
Brain endothelium
Di−telencephalon
Hindbrain
Mesencephalon−MHB
Di−mesencephalon excitatory neurons
Di−mesencephalon inhibitory neurons
Weight
1
0
b
D1-muscle
Myocytes
Endothelium
White blood cells
Connective tissue progenitors
Definitive erythroid cells
0.0 0.5 1.0
D2-craniofacial primordium
Myocytes
Osteoblast progenitors B
Endothelium
Branchial arch epithelium
Olfactory epithelium
0.0 0.4 0.8
D3-endochondral bone
Myocytes
Endothelium
White blood cells
Connective tissue progenitors
Osteoblast progenitors B
0.0 0.5 0.9
D4-primordium of chondrocranium
Myocytes
Osteoblast progenitors B
Connective tissue progenitors
Endothelium
Pre−epidermal keratinocytes
0.0 0.2 0.4
D5-visceral organ with smooth muscle
Endothelium
Definitive erythroid cells
Splanchnic mesoderm
Myocytes
Amniochorionic mesoderm A
0.0 0.3
D6-hepatic parenchyma
Definitive erythroid cells
Hepatocytes
Megakaryocytes
White blood cells
Splanchnic mesoderm
0.0 0.5 1.0
D7-midbrain
Neuron progenitor cells
Inhibitory interneurons
Noradrenergic neurons
Spinal cord excitatory neurons
Di−mesencephalon inhibitory neurons
0.0 0.1
D8-limbs and genital tubercle
Endothelium
Limb mesenchyme progenitors
Myocytes
Definitive erythroid cells
Pre−epidermal keratinocytes
0.0 0.3 0.6
D9-cartilage matrix
Myocytes
Endothelium
Limb mesenchyme progenitors
Osteoblast progenitors B
Connective tissue progenitors
0.0 0.5 0.9
D10-mucosa and primordium
Myocytes
Endothelium
Definitive erythroid cells
Noradrenergic neurons
Splanchnic mesoderm
0.0 0.4
D11-telencephalon
Inhibitory interneurons
Di−telencephalon
Intermediate progenitor cells
Neuron progenitor cells
Mesencephalon−MHB
0.0 0.2 0.5
D12-cephalic mesenchyme and blood vessels
Early chondrocytes
White blood cells
Brain endothelium
Myocytes
Endothelium
0.0 0.4
D13-ganglion
Neural crest (PNS neurons)
Myocytes
Endothelium
Early chondrocytes
Neural crest (PNS glia)
0.0 0.3 0.5
D14-neopallium
Di−telencephalon
Intermediate progenitor cells
Brain endothelium
Early chondrocytes
Osteoblast progenitors B
0.0 0.4 0.8
D15-skin
Pre−epidermal keratinocytes
Myocytes
Endothelium
Osteoblast progenitors B
Definitive erythroid cells
0.0 0.3 0.6
D16-medulla oblongata and spinal cord
Neuron progenitor cells
Spinal cord excitatory neurons
Spinal cord inhibitory neurons
Myocytes
Motor neurons
0.0 0.1
Fraction
D17-ependyma
Mesencephalon−MHB
Neuron progenitor cells
Spinal cord (dorsal)
Hindbrain
Di−telencephalon
0.0 0.2
Fraction
D18-mesentery and septum transversum
Splanchnic mesoderm
Definitive erythroid cells
White blood cells
Endothelium
Myocytes
0.0 0.3 0.7
Fraction
D19-heart
Cardiomyocytes
Endothelium
Myocytes
Splanchnic mesoderm
Definitive erythroid cells
0.0 0.4 0.8
Fraction

Supplementary Fig.13| Cell type compositions in spatial domains across the embryo. (**a**) Spatial visualization of deconvoluted weights of definitive erythroid cells, liver endothelium, megakaryocytes, amniochorionic mesoderm A and brain related cell type after spatial mapping. (**b**) The top five abundant cell types that localized in each spatial domain. The cell types were selected by the deconvoluted weights greater than 0.05. Source data are provided as a Source Data file.

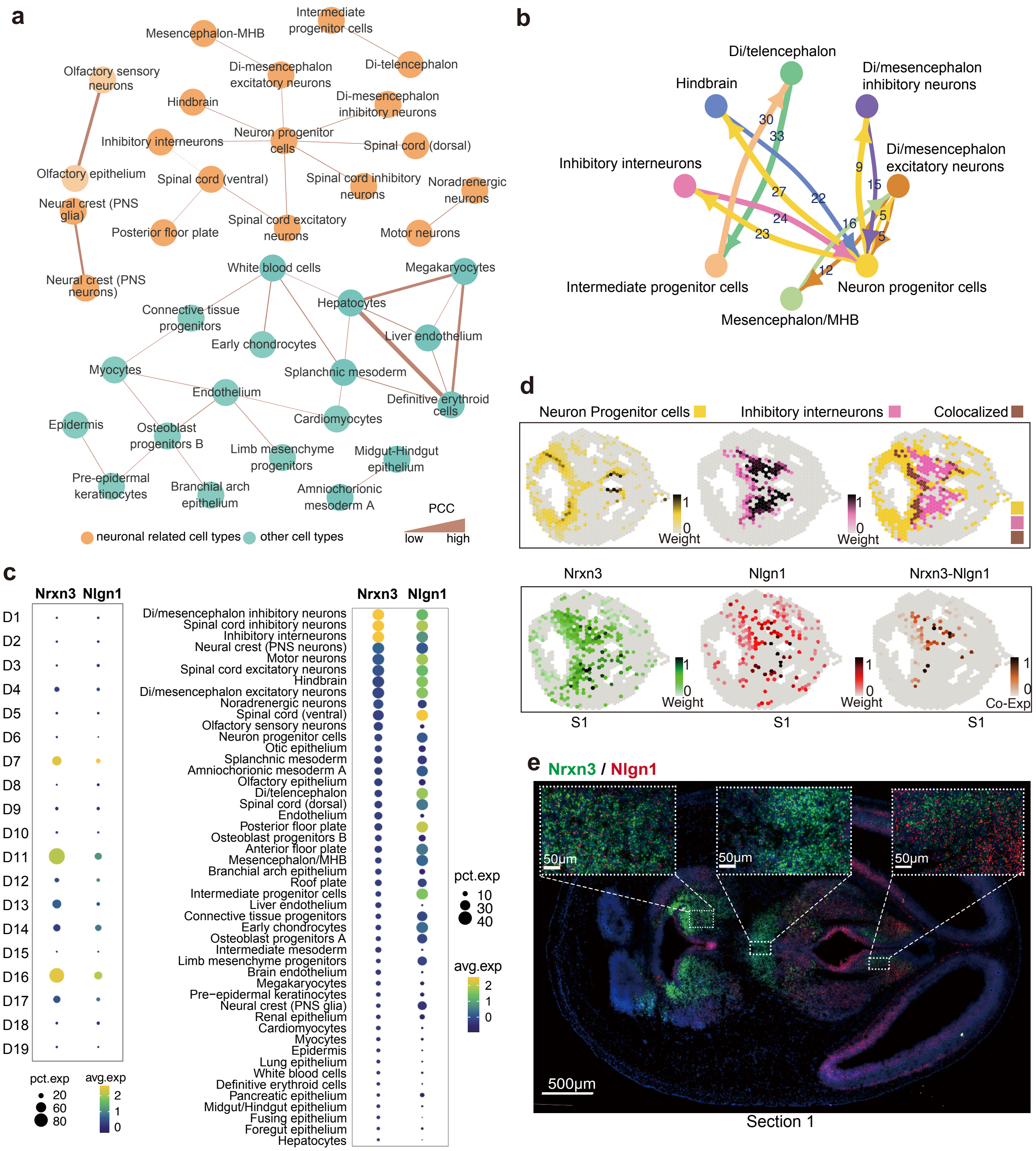

a
Intermediate progenitor cells
Mesencephalon-MHB
Di-telencephalon
Olfactory sensory neurons
Di-mesencephalon excitatory neurons
Hindbrain
Di-mesencephalon inhibitory neurons
Inhibitory interneurons
Neuron progenitor cells
Spinal cord (dorsal)
Olfactory epithelium
Spinal cord (ventral)
Spinal cord inhibitory neurons
Noradrenergic neurons
Neural crest (PNS glia)
Posterior floor plate
Spinal cord excitatory neurons
Motor neurons
Neural crest (PNS neurons)
White blood cells
Megakaryocytes
Connective tissue progenitors
Hepatocytes
Early chondrocytes
Liver endothelium
Myocytes
Splanchnic mesoderm
Endothelium
Definitive erythroid cells
Epidermis
Osteoblast progenitors B
Cardiomyocytes
Limb mesenchyme progenitors
Midgut-Hindgut epithelium
Pre-epidermal keratinocytes
Branchial arch epithelium
Amniochorionic mesoderm A
PCC
low
high
neuronal related cell types
other cell types
b
Di/telencephalon
Hindbrain
Di/mesencephalon inhibitory neurons
Inhibitory interneurons
Di/mesencephalon excitatory neurons
Intermediate progenitor cells
Neuron progenitor cells
Mesencephalon/MHB
30
33
9
15
27
22
24
16
23
5
5
12
c
Nrxn3
Nlgn1
D1
D2
D3
D4
D5
D6
D7
D8
D9
D10
D11
D12
D13
D14
D15
D16
D17
D18
D19
pct.exp
20
60
80
avg.exp
2
1
0
Di/mesencephalon inhibitory neurons
Spinal cord inhibitory neurons
Inhibitory interneurons
Neural crest (PNS neurons)
Motor neurons
Spinal cord excitatory neurons
Hindbrain
Di/mesencephalon excitatory neurons
Noradrenergic neurons
Spinal cord (ventral)
Olfactory sensory neurons
Neuron progenitor cells
Otic epithelium
Splanchnic mesoderm
Amniochorionic mesoderm A
Olfactory epithelium
Di/telencephalon
Spinal cord (dorsal)
Endothelium
Posterior floor plate
Osteoblast progenitors B
Anterior floor plate
Mesencephalon/MHB
Branchial arch epithelium
Roof plate
Intermediate progenitor cells
Liver endothelium
Connective tissue progenitors
Early chondrocytes
Osteoblast progenitors A
Intermediate mesoderm
Limb mesenchyme progenitors
Brain endothelium
Megakaryocytes
Pre−epidermal keratinocytes
Neural crest (PNS glia)
Renal epithelium
Cardiomyocytes
Myocytes
Epidermis
Lung epithelium
White blood cells
Definitive erythroid cells
Pancreatic epithelium
Midgut/Hindgut epithelium
Fusing epithelium
Foregut epithelium
Hepatocytes
pct.exp
10
30
40
avg.exp
2
1
0
d
Neuron Progenitor cells
Inhibitory interneurons
Colocalized
1
0
Weight
Nrxn3
Nlgn1
Nrxn3-Nlgn1
Co-Exp
S1
e
Nrxn3 / Nlgn1
50μm
500μm
Section 1

Supplementary Fig.14| Cell-cell communication based on spatial and TOME single cell RNA-seq data.

(a) Network plot showing the colocalized cell type pairs. The edge width is proportional to the indicated PCC of the connected cell type pairs. The orange color indicated the brain related cell types, and the green colors indicated all other cell types. (**b**) The interaction network of significant L-Rs between pair of two cell populations from spots co-occurred brain related cell types by STcomm. The edge width is proportional to the indicated number of L-Rs. (**c**) Dot plots showing the expression distribution of *Nrxn3* and *Nlgn1* at different spatial domains in our ST data (left) and at different cell populations in TOME single-cell data at E13.5 (right). The dot color and size represent the average expression and percentage of spots in each group. (**d**) Spatial plots showing the spatial distribution (color intensity) and colocalization of neuron progenitor cells and inhibitory interneurons according to predicted weights by deconvolution in S1 (Top panel). The bottom plots showing the spatial distribution of expression and co-expression of L-Rs Nrxn3 and Nlgn1 in S1, which is similar to Fig.6i, but illustrated on the embryo tissue of section 1. (**e**) *Nrxn3* and *Nlgn1* spatial expression pattern detected by RNAScope in brain tissue section matched to section 1 in (**d**), n = 3. White dashed box showing the staining of *Nrxn3* and *Nlgn1* in spatial proximity cells. Source data are provided as a Source Data file.
