# Supplementary material for: Three-dimensional molecular architecture of mouse organogenesis
Source: Nat Commun. 2023 Jul 31;14:4599. doi: 10.1038/s41467-023-40155-7 (PMC10390492; doi:10.1038/s41467-023-40155-7)
Supplement: Supplementary file 3 — Description of Additional Supplementary Files [file 41467_2023_40155_MOESM3_ESM.pdf]

Description of Additional Supplementary Files

Supplementary Data 1: The differentially expressed genes identified in all major spatial domains with a log fold change > 0.25, adjust P threshold of 0.05. Statistical significance was calculated by two-sided Wilcoxon Rank Sum test with Bonferroni correction.

Supplementary Data 2: The ranked regulon specific score (RSS) in all major spatial domains.

Supplementary Data 3: The literature reviewing of selected top ranked regulons in spatial domains.

Supplementary Data 4: The differentially expressed genes identified in 10 subclusters of spatial domain 5-visceral organ with smooth muscle with a log fold change > 0.25, adjust P threshold of 0.05. Statistical significance was calculated by two-sided Wilcoxon Rank Sum test with Bonferroni correction.

Supplementary Data 5: The regulon specific score (RSS) of top 5 regulons in 10 subclusters of spatial domain 5-visceral organ with smooth muscle.

Supplementary Data 6: The differentially expressed genes identified in 4 subregions of D19-heart with a log fold change > 0.25, adjust P threshold of 0.05. Statistical significance was calculated by two-sided Wilcoxon Rank Sum test with Bonferroni correction.

Supplementary Data 7: The top Anterior-Posterior axis related genes identified in spinal cord region (see Method and tatistical significance was computed from likelihood ratio tests determined by Monocle2).

Supplementary Data 8: The differentially expressed genes identified in Dorsal, Medial and Ventral region of spinal cord. Statistical significance was calculated by two-sided Wilcoxon Rank Sum test with Bonferroni correction.

Supplementary Data 9: The predicted deconvolution weight (proportion) of confident cell types for each spot pixel by RCTD on multi-mode.

Supplementary Data 10: The spatial resolved cell-cell communication by integrating the spatial atlas and single-cell data with STcomm (see Methods and p-values were calculated from one-sided permutation test by CellChat).

Supplementary Movie 1: illustration of spatial domains in a 3D embryo model.
