# Supplementary figures and images for: Three-dimensional molecular architecture of mouse organogenesis
Source: Nat Commun. 2023 Jul 31;14:4599. doi: 10.1038/s41467-023-40155-7 (PMC10390492; doi:10.1038/s41467-023-40155-7)

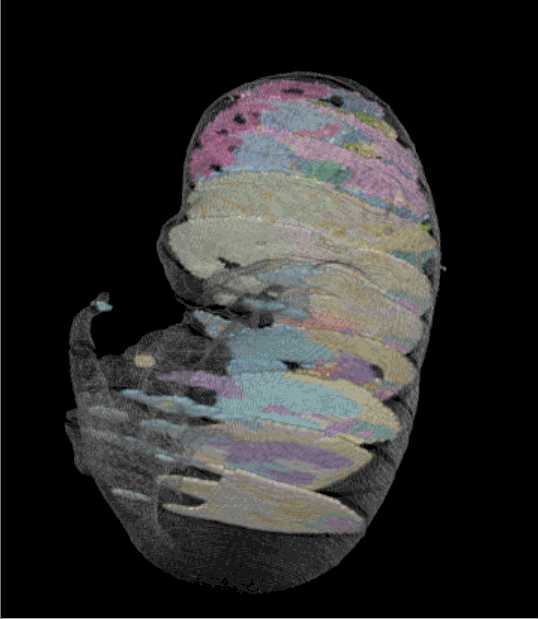

Supplement: Supplementary file 14 — Supplementary Movie 1 [file 41467_2023_40155_MOESM14_ESM.gif]
