# Supplementary material for: Three-dimensional molecular architecture of mouse organogenesis
Source: Nat Commun. 2023 Jul 31;14:4599. doi: 10.1038/s41467-023-40155-7 (PMC10390492; doi:10.1038/s41467-023-40155-7)
Supplement: Supplementary file 15 — Reporting Summary [file 41467_2023_40155_MOESM15_ESM.pdf]

nature portfolio

Corresponding author(s): Guangdun Peng, Shengbao Suo, Guizhong Cui

Last updated by author(s): June 7, 2023

Reporting Summary

Nature Portfolio wishes to improve the reproducibility of the work that we publish. This form provides structure for consistency and transparency in reporting. For further information on Nature Portfolio policies, see our Editorial Policies and the Editorial Policy Checklist.

Statistics

For all statistical analyses, confirm that the following items are present in the figure legend, table legend, main text, or Methods section.

n/a	Confirmed	
☐	☒	The exact sample size (*n*) for each experimental group/condition, given as a discrete number and unit of measurement
☐	☒	A statement on whether measurements were taken from distinct samples or whether the same sample was measured repeatedly
☐	☒	The statistical test(s) used AND whether they are one- or two-sided<br>*Only common tests should be described solely by name; describe more complex techniques in the Methods section.*
☒	☐	A description of all covariates tested
☐	☒	A description of any assumptions or corrections, such as tests of normality and adjustment for multiple comparisons
☐	☒	A full description of the statistical parameters including central tendency (e.g. means) or other basic estimates (e.g. regression coefficient) AND variation (e.g. standard deviation) or associated estimates of uncertainty (e.g. confidence intervals)
☐	☒	For null hypothesis testing, the test statistic (e.g. F, t, r) with confidence intervals, effect sizes, degrees of freedom and P value noted<br>*Give P values as exact values whenever suitable.*
☒	☐	For Bayesian analysis, information on the choice of priors and Markov chain Monte Carlo settings
☒	☐	For hierarchical and complex designs, identification of the appropriate level for tests and full reporting of outcomes
☐	☒	Estimates of effect sizes (e.g. Cohen's *d*, Pearson's *r*), indicating how they were calculated

Our web collection on statistics for biologists contains articles on many of the points above.

Software and code

Policy information about availability of computer code

Data collection	Image acquisition: Olympus SZX16 was used for obtain bright-field images of whole-mount embryo and colorimetric in-situ hybridization images; Zeiss Axio Observer 7 and Olympus VS200 were used to obtain cresyl violet images of cryosection for spatial transcripomic sequencing; Olympus VS200 was used for obtain fluorescence-field images of RNAScope on crysections. Next generation sequencing acquisition: The cDNA libraries were sequenced on Ilumina Novaseq 6000 system with paired-end 150bp reads, aiming for 100 k raw reads per spots.
Data analysis	Software, packages and algorithms version are listed here:<br>fastp: 0.21.0<br>Space Ranger: 1.0.0<br>Loupe Browser: 4.0.0<br>Seurat: 3.2 and 4.0.5<br>Giotto: 1.2<br>clusterProfiler: 3.14.3<br>pySCENIC: 0.10.3<br>Cytoscape: 3.9.1<br>Monocle: 2.18.0<br>AUCell: 1.8.0<br>spacexr: 2.0.0<br>CellChat: 1.4.0<br>STcomm: https://doi.org/10.5281/zenodo.7988217<br>Shiny: 1.7.4

shinythemes: 1.2.0
ComplexHeatmap: 2.6.2
circlize: 0.4.15
plotly: 4.10.1
ImageJ: 1.53c
MATLAB: R2020a
Adobe Photoshop: CC2019

For manuscripts utilizing custom algorithms or software that are central to the research but not yet described in published literature, software must be made available to editors and reviewers. We strongly encourage code deposition in a community repository (e.g. GitHub). See the Nature Portfolio guidelines for submitting code & software for further information.

Data

Policy information about availability of data

All manuscripts must include a data availability statement. This statement should provide the following information, where applicable:

- Accession codes, unique identifiers, or web links for publicly available datasets
- A description of any restrictions on data availability
- For clinical datasets or third party data, please ensure that the statement adheres to our policy

The mouse organogenesis spatial transcriptomic raw data and corresponding section images generated in this study have been deposited in the NODE (The National Omics Data Encyclopedia) database under accession code OEP003721 (https://www.biosino.org/node/project/detail/OEP003721) and can be explored at the web portal (http://most.ccla.ac.cn). The MOSTA data at stage of E13.5 used in this study are available in the CNGB database under accession code CNP0001543 (https://db.cngb.org/search/project/CNP0001543/) and the processed data are available at https://db.cngb.org/stomics/mosta. The single cell dataset of heart for used in deconvolution analysis used in this study are available in the GEO under accession code GSE193346 (https://www.ncbi.nlm.nih.gov/geo/query/acc.cgi?acc=GSE193346), and TOME data used in this study are available under accession code GSE186068 (https://www.ncbi.nlm.nih.gov/geo/query/acc.cgi?acc=GSE186068) and the processed data are available at http://tome.gs.washington.edu/. Source data are provided with this paper.

Research involving human participants, their data, or biological material

Policy information about studies with human participants or human data. See also policy information about sex, gender (identity/presentation), and sexual orientation and race, ethnicity and racism.

Reporting on sex and gender	N/A
Reporting on race, ethnicity, or other socially relevant groupings	N/A
Population characteristics	N/A
Recruitment	N/A
Ethics oversight	N/A

Note that full information on the approval of the study protocol must also be provided in the manuscript.

Field-specific reporting

Please select the one below that is the best fit for your research. If you are not sure, read the appropriate sections before making your selection.

☒ Life sciences ☐ Behavioural & social sciences ☐ Ecological, evolutionary & environmental sciences

For a reference copy of the document with all sections, see nature.com/documents/nr-reporting-summary-flat.pdf

Life sciences study design

All studies must disclose on these points even when the disclosure is negative.

Sample size	A total of 10 sections along craniocaudal axis at 10um thichness (about every 100 sections apart from each other) from a male embryo (E1)were collected for spatial tanscriptomic analysis as a reference atlas, with 16418 high-quality spots profiled. We also selected two sections from head part of the repeat male embryo (E2) with similar morphology to the reference section 1 and section2 in E1. And four sections were collected from the body part of the repeat female embryo (E3), which were about half away from the section 6 to 9. In total, there were 16 Visium libraries of E13.5 embryos.
Data exclusions	The expression datasets were filtered with cut-offs at a minimum of 1,000 detected genes and a maximum of 10% mitochondrial counts per spot for 10 sections of E1 and E3, except for section 6.5 (F6.5) of E3. A minimum of 500 detected genes were applied for F6.5 section.
Replication	Three replicates at E13.5 are collected with two male embryos and one female embryo.
Randomization	Mid-gestation organogenesis stage embryonic day 13.5, E13.5) embryos were collected from C57/BL6 mice, and embryos images were taken

Randomization	for recording and confirmation of development staging. For every replicates, the whole embryo was serially cryo-sectioned into about 1000 sections along craniocaudal axis at 10um thickness. And we selected slices with an average interval of 100 sections. These selected sections were used for spatial transcriptomic analysis by modified 10X Genomics Visium platform.
Blinding	Not applicable since no specific grouping.

Reporting for specific materials, systems and methods

We require information from authors about some types of materials, experimental systems and methods used in many studies. Here, indicate whether each material, system or method listed is relevant to your study. If you are not sure if a list item applies to your research, read the appropriate section before selecting a response.

Materials & experimental systems

n/a	Involved in the study
☐	☒ Antibodies
☒	☐ Eukaryotic cell lines
☒	☐ Palaeontology and archaeology
☐	☒ Animals and other organisms
☒	☐ Clinical data
☒	☐ Dual use research of concern
☒	☐ Plants

Methods

n/a	Involved in the study
☒	☐ ChIP-seq
☒	☐ Flow cytometry
☒	☐ MRI-based neuroimaging

Antibodies

Antibodies used	Anti-DIG antibody conjugated to alkaline phosphatase (1:2000, Sigma-Aldrich, 11093274910).
Validation	Anti-DIG antibody conjugated to alkaline phosphatase (1:2000, Sigma-Aldrich, 11093274910). Validation: Komatsu, Y., Kishigami, S. & Mishina, Y. In situ hybridization methods for mouse whole mounts and tissue sections with and without additional β-galactosidase staining. Methods Mol. Biol. 1092, 1–15 (2014).

Animals and other research organisms

Policy information about studies involving animals; ARRIVE guidelines recommended for reporting animal research, and Sex and Gender in Research

Laboratory animals	Species: mouse. strain: C57BL/6JGpt. Gpt: ICR. Sex: male and female. Ages: embryonic days at E13.5. All animals were housed with a standard light/dark cycle and availability of food and water. The ambient temperature was 24~26 ℃ and humidity was 50%~60%.
Wild animals	The study did not involve wild animals.
Reporting on sex	Male (n=2) and female (n=1) mouse embryos were used to generate spatial datasets.
Field-collected samples	The study did not involve samples collected from the field.
Ethics oversight	All animal procedures conducted in this study were approved by the Institutional Animal Care and Use Committee of Guangzhou Institutes of Biomedicine and Health (GIBH), Guangdong.

Note that full information on the approval of the study protocol must also be provided in the manuscript.
